# Experimental Study of Mechanical Properties and Impact-Induced Reaction Characteristics of PTFE/Al/CuO Reactive Materials

**DOI:** 10.3390/ma13010066

**Published:** 2019-12-21

**Authors:** Jingyuan Zhou, Liangliang Ding, Wenhui Tang, Xianwen Ran

**Affiliations:** College of Liberal Arts and Sciences, National University of Defense Technology, Changsha 410073, China; zhoujingyuan19@nudt.edu.cn (J.Z.); ranxianwen@nudt.edu.cn (X.R.)

**Keywords:** metal/fluoropolymer material, reactive materials, numerical simulation, drop hammer test

## Abstract

Metal/fluoropolymer materials are typical reactive materials. Polytetrafluoroethylene (PTFE)/Al/CuO reactive materials were studied in this research. Scanning electron microscopy (SEM), quasi-static compression, the Split Hopkinson pressure bar test, and the drop hammer test were used to study the mechanical properties and induced reaction characteristics of the reactive materials with different Al/CuO thermite contents and different particle sizes. SEM images of the samples demonstrate that the reactive materials were mixed evenly. The compression test results show that, if the particle size of PTFE was too small, the strength of reactive materials after sintering was reduced. After sintering, with the increase in the content of Al/CuO thermite, the strength of the micro-sized PTFE/Al/CuO firstly increased and then decreased. The Johnson–Cook constitutive model can be used to characterize the reactive materials, and the parameters of the Johnson–Cook constitutive model of No. 3 reactive materials (PTFE/Al:Al/CuO = 3:1) were obtained. The reliability of the parameters was verified by numerical simulation. Drop hammer tests show that the addition of Al/CuO aluminothermic materials or the use of nano-sized PTFE/Al reactive materials can significantly improve the sensitivity of the material. The research in this paper can provide a reference for the preparation, transportation, storage, and application of reactive materials.

## 1. Introduction

In the early 1970s, two scientists serving the United States (US) Office of Naval Research (ONR), Willis and Holt [1,2], used light gas guns to study the mechanical behavior of materials under high-speed impact. They found that Polytetrafluoroethylene (PTFE)/Al composites release energy under high-speed impact conditions. Such materials that release energy under impact conditions are called reactive materials. In the following 40 years, a lot of research was done on reactive materials. These materials can be polymer/metal or metal/metal composite mixtures [3]. The reactive material is inert under normal conditions, and its stability is higher than that of conventional explosives [4]. Reaction occurs only under high-speed impact or high-strain-rate loading conditions [5]. The energy density of the reactive material is very high. For example, the theoretical energy density of the PTFE/Al reactive material is more than three times that of the same mass of Trinitrotoluene (TNT) [6]. Due to the many advantages of reactive materials, their application in many fields, especially the military field, was studied deeply [7,8,9].

Among the metal/fluoropolymer reactive materials, the PTFE/Al composite is the most studied. Since it was found that the density of PTFE reactive materials is low and their strength is not sufficient, many researchers tried increasing the strength of PTFE/Al by adding W or Ni elements [10,11,12,13,14,15,16,17]. It was found that adding a high-strength metal material, such as W, with a similar particle size to the PTFE/Al reactive material, can effectively increase the strength of the reactive materials. Cai et al. [12] used a drop hammer to find that the W particle–PTFE interface separation provided initiation and propagation of cracks. In general, the addition of W can increase the strength of the reactive material but reduce the energy density. Thus, people considered adding materials that can release energy, such as Ni. After adding Ni to PTFE/Al, Wu et al. [16] found that Ni makes the reactive material brittle, but it can increase the strain-hardening modulus and compressive strength of the material, while the heat released by the material also increased. However, the energy density of PTFE/Al reactive material is very high, and the energy of PTFE/Al reactive material cannot be significantly increased by adding Ni. If the energy release efficiency of the PTFE reactive materials can be effectively improved, it will be beneficial for their application. PTFE filler metal is a very common type of engineering material. The C–F bonds in PTFE are usually stable and do not react with metals. Under high-temperature conditions, PTFE rapidly decomposes into small-molecule fluorides and undergoes a rapid exothermic reaction with active metals [18]. Experiments by Ames [19] showed that, when the PTFE/Al reactive material impacts the target at a velocity of 1.2 km/s, the energy release efficiency still does not exceed 20%. Therefore, one of the biggest problems with PTFE/Al reactive materials is that the energy release efficiency is too low. Some researchers considered adding materials such as Fe_2_O_3_, Mg, TiH_2_, or MnO_2_ [20,21,22,23,24]. These materials react more readily with Al or PTFE and release a large amount of heat, which promotes the reaction of PTFE/Al. It was also found that the compressive strength of the PTFE/Al reactive materials is improved after adding appropriate amounts of these materials. This may be a good idea to appropriately reduce the reaction conditions of the reactive materials, enabling a better heat release performance.

The idea of adding a material that promotes the reaction should be effective in improving the performance of the reactive materials. The reaction rate of Al/CuO thermite is one of the fastest among known thermites. Its combustion velocity can be as high as 2400 m/s [25]. Considering the phase change, the adiabatic reaction temperature of Al/CuO thermite can reach 2834 °C, and the reaction heat of Al/CuO thermite can reach 4077 J/g [26]. The sensitivity of Al/CuO thermite is also very high. These factors are very beneficial to promote the occurrence and spread of the reaction and improve the efficiency of the reaction. The density of CuO is 6.4 g/cm^3^, and its strength is also high as a metal oxide, which can be used to improve the mechanical properties of reactive materials.

Based on the above research, we studied the energy release characteristics of reactive materials [27]. The mechanical properties and impact-induced reaction characteristics of PTFE/Al reactive materials with the addition of CuO were studied in this paper. Scanning electron microscopy, a universal material testing machine, a Split Hopkinson pressure bar (SHPB) device, and a drop hammer device were used to test and analyze the PTFE/Al/CuO reactive materials. These test methods are commonly used to test reactive materials [5,15,16,23]. Scanning electron microscopy was used to observe the microstructure of the reactive material. Quasi-static and dynamic compression properties of reactive materials were tested using the universal material testing machine and the SHPB device. Then, the Johnson–Cook model parameters of reactive materials were fitted and verified by numerical simulation. The drop hammer device was used to study the impact sensitivity of the reactive material. The research in this paper can provide a reference for the preparation, transportation, storage, and application of reactive materials.

## 2. Materials and Experimental Methods

### 2.1. Sample Preparation Method

The material preparation process mainly adopted the cold pressing and vacuum sintering method, which is similar to the general powder material forming process. The preparation process of the reactive materials was mainly divided into the following steps:

(1) Formulation of the reactive materials. The main reaction equations for PTFE and Al in an inert gas were as follows:

(1) $${4\mathrm{Al}+3C}_{2}F_{4}{=4\mathrm{AlF}}_{3}+6C$$

The reaction equation between Al and CuO was as follows:

(2) $${3\mathrm{Al}+3\mathrm{CuO}=\mathrm{Al}}_{2}O_{3}+3\mathrm{Cu}$$

According to the reaction equation, the mass ratio of Al to PTFE was 26.5:73.5, and that of Al to CuO was 18.5:81.5. Firstly, PTFE/Al and Al/CuO were arranged based on their reaction mass ratios. Then, PTFE/Al and Al/CuO were mixed together in different proportions to form different material formulations. The formulations of the reactive materials are shown in Table 1.

(2) Uniform mixing of powders. The main raw materials for reactive materials in this paper included Al (10 μm and 50 nm), PTFE (35 μm and 350 nm), and CuO (50 nm) powders, as shown in Table 2. In order to have better energy release efficiency, only nano-sized Al/CuO thermite was selected.

Since PTFE is an extremely hydrophobic organic high-molecular polymer with a poor affinity for metals such as Al, it was necessary to optimize its surface first. At this point, the coupling agent could be used to improve the contact problem between the composite materials [6]. A coupling agent is a kind of substance with two different functional groups. One is a pro-inorganic group that is susceptible to chemical reactions with inorganic surfaces. The other is an organophilic group that is capable of chemically reacting with a synthetic resin or other polymer by forming a hydrogen bond therein. A bonding layer can be formed between the inorganic substance and the organic substance to improve the performance of the composite material. According to our previous research, silane coupling agent had a better coupling effect on PTFE/Al [27]. The detailed operation method was as follows:

(a) The coupling agent was dissolved in absolute ethanol, and the mass of the coupling agent was 1% of the metal powder.
(b) The metal powder was placed into organic solvent for 1 h, and it was heated properly and stirred until the organic solvent evaporated completely.
(c) The metal powder was thoroughly dried in a vacuum oven.
(d) PTFE and the surface-treated metal powder were placed in absolute ethanol and stirred for 10 h. Afterward, the mixture was properly heated to completely volatilize organic solvents. Finally, the material was thoroughly dried using a vacuum drying oven.

(3) Powder pressing forming. An appropriate quantity of powder material was placed into the mold and pressed for 1 min at 200 MPa. After removing the sample, it was allowed to stand for 24 h to eliminate internal stress in the material.

(4) Material sintering. The PTFE material as the matrix determines the main mechanical properties of the reactive material. Through the sintering process, the PTFE can be melted and recrystallized, and the interface between particles disappears due to diffusion. PTFE turns from particles into a dense continuum, and the strength of PTFE is greatly improved [28]. After sintering, PTFE encapsulates the active metal. It can isolate the metal from the external environment, thereby reducing the chance of the active metal being oxidized. In order to avoid the reaction of the reactive material, sintering needs to be carried out in a vacuum or inert gas environment.

**Figure 1:**
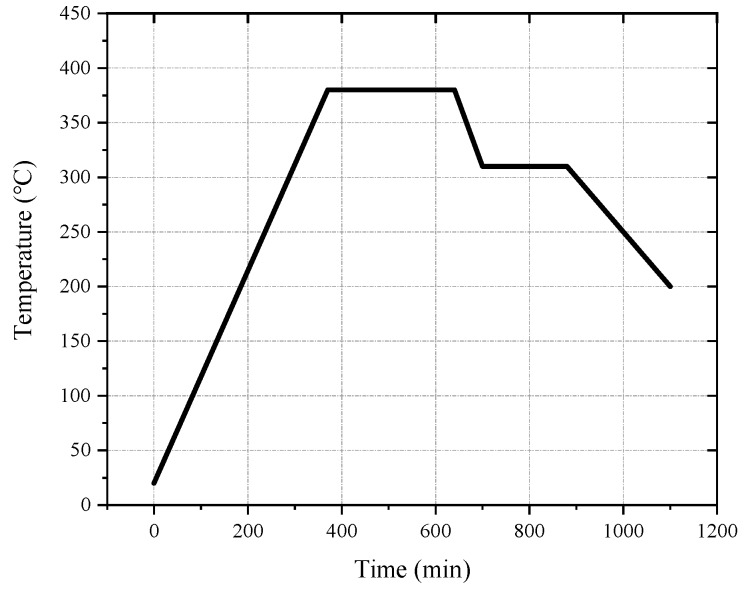
The sintering temperature curve of reactive materials.

The melting temperature of PTFE is 327 °C. When the temperature exceeds 400 °C, PTFE begins to decompose. When the temperature exceeds 500 °C, PTFE decomposes rapidly. It is generally assumed that the optimum sintering temperature is 380 °C [6,28,29,30]. Based on the size of the sample and referring to other research [15,17,30], the design sintering process was as follows: the oven temperature was ramped up to 380 °C at a rate of approximately 55 °C/h. Following this, the sintering temperature was maintained at 380 °C for 1–6 h, depending on the size of the sample. Then, the temperature was reduced to 315 °C at a rate of 55 °C/h and maintained for 2 h. Finally, the sample was cooled to room temperature at a rate not exceeding 55 °C/h. The sintering curve is shown in Figure 1.

**Figure 2:**
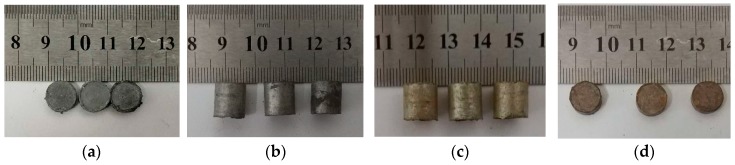
Test samples: (**a**,**b**) reactive material without added Al/CuO thermite; (**c**,**d**) reactive materials containing Al/CuO thermite.

The samples are shown in Figure 2. We used the same sample from a previous study [27]. It is obvious that the reactive material containing thermite was reddish in color. This indicates that a small amount of CuO underwent a reduction reaction during the sintering process to form a copper element.

**Figure 3:**
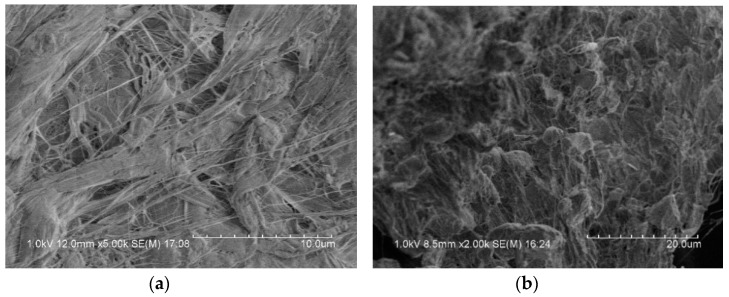
Scanning electron microscope image of reactive materials; (**a**) PTFE fiber; (**b**) PTFE coated metal particles.

Figure 3 is a microscopic image of the surface of the reactive material under an electron microscope. It can be observed that some bright spheres were included in the matrix of PTFE, which were Al particles. CuO particles were very small and, therefore, difficult to observe. The metal particles were coated in the PTFE matrix, and the binding was relatively tight, but a small number of pores could be seen in some places.

### 2.2. Quasi-Static and Dynamic Mechanics Testing

Quasi-static compression tests were performed in a standard laboratory environment (23 ± 2 °C, 30–40% relative humidity) using an MTS universal testing machine. The dimensions of the cylindrical samples were ∅10 ± 0.4 mm × 10 ± 0.2 mm (∅10 mm indicates diameter, ×10 mm indicates thickness), and the strain rate of the quasi-static test was 10^−3^ s^−1^. According to the size of the sample, the specific compression speed was 0.6 mm/min. Before the test, both ends of the sample were smoothed to ensure that the force was uniform. The engineering stress and engineering strain curves could be obtained directly in the instrument. Considering that the diameter of the specimen increases during compression, the engineering stress $\sigma_{eng}$ and engineering strain $\varepsilon_{eng}$ directly measured by the test needed to be converted using the formulas below. The stress and strain values of compression are shown as positive numbers.

(3) $$\varepsilon =-\ln(1-\varepsilon_{eng})$$

(4) $$\sigma =(1-\varepsilon_{eng})\sigma_{eng}$$

**Figure 4:**
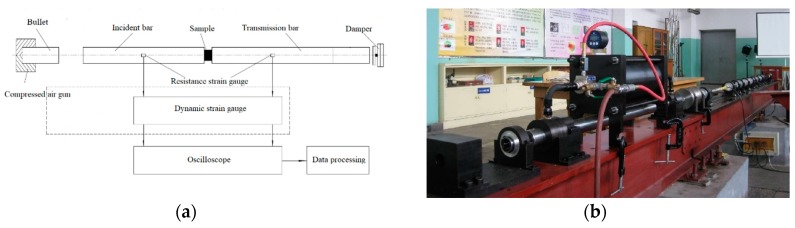
Split Hopkinson pressure bar (SHPB) system. (**a**) SHPB schematic; (**b**) actual device.

The dynamic compression was tested using the Split Hopkinson pressure bar (SHPB) system [31]. The SHPB test system is an indispensable experimental tool for studying the response of medium- and high-strain-rate materials. It is a classic method for measuring the medium- and high-strain-rate impact compression behavior, and it is also a basic experiment for obtaining the dynamic constitutive equation of materials. The SHPB experimental technique is based primarily on two fundamental postulates: (1) one-dimensional elastic stress wave theory—it is necessary to ensure that the sample is in a one-dimensional stress state in the test; (2) with the assumption that the stress and strain of the samples are uniformly distributed along the axial direction. The SHPB experimental device is shown in Figure 4.

The test process was as follows: a compression pulse signal was generated by the bullet impacting the incident bar at high speed, which caused the sample to interact with the incident bar and the transmission bar. The strain signals in the incident bar and the transmission bar were measured by strain gauges attached to the bar. Based on one-dimensional theory, the stress σ, strain ε, and strain rate $\dot{\varepsilon }$ of the sample could be obtained by the following formulas:

(5) $$\sigma =\frac{AE}{A_{0}}\varepsilon_{t},$$

(6) $$\varepsilon =-\frac{2c_{0}}{l_{0}}\int_{0}^{t}\varepsilon_{r}dt,$$

(7) $$\dot{\varepsilon }=-\frac{2c_{0}}{l_{0}}\varepsilon_{r},$$

where *ε_t_* and *ε_r_* are the transmission strain and the reflection strain, *A* and *A*_0_ represent the cross-sectional area of the compression bar and the sample, *c*_0_ is the speed of sound in the bar, *l*_0_ is the initial length of the sample, and *E* is the elastic modulus of the bar.

The dimensions of the samples used in SHPB test in this paper were ∅10 ± 0.4 mm × 3 ± 0.2 mm. The bar used in the test was an Al bar with a diameter of 20 mm. The length of the bullet was 250 mm, while that of the incident bar and transmitted bar was 2100 mm. Tests were also performed in a standard laboratory environment.

### 2.3. Drop Hammer Test Method

**Figure 5:**
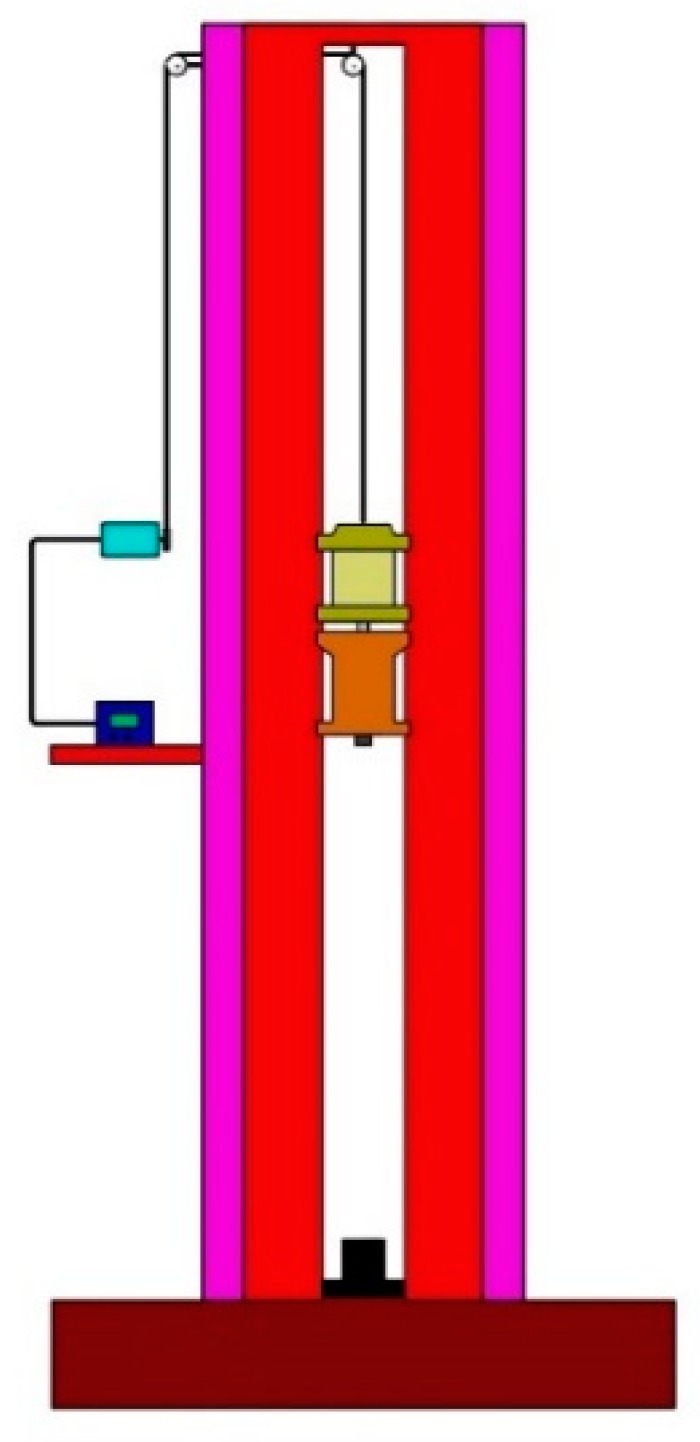
Drop hammer test system.

The drop hammer test is generally used to obtain the characteristics of a sample under impact by heavy objects. It is often used to test the impact sensitivity of energetic materials. High-speed cameras are usually required to record the test process. The drop hammer device is shown in Figure 5. The mass of the hammer was 10 kg, and the hammer head was a cylinder with a diameter of 30 mm. According to requirements, the impact surface of the drop hammer was free of unevenness and other serious mechanical damage.

There are several common methods to test the mechanical sensitivity of materials using drop hammer test. The special height method is used in some special cases to analyze the height at which the probability of material reaction is 50%. The upper and lower limit method is used to measure the critical height at which 100% of the energetic materials react or do not react. The percent explosion method uses a fixed drop hammer and fixed height to measure the percentage reaction of energetic material.

In this paper, the lower limit method was used to analyze the impact sensitivity of materials. That is to say, we needed to measure the maximum drop hammer height at which the reactive material did not react 100%. Firstly, the reaction threshold height of the sample was estimated by the previous test. Then, we selected a height close to the threshold height to start the drop hammer test. If there was a reaction, we moved the drop hammer down by 1 cm. If there was no reaction, we let the drop hammer rise by 1 cm. Each material needed to be tested at least 10 times to ensure the reliability of the results, and we set up a high-speed camera (Phantom V710) to record the test process.

**Figure 6:**
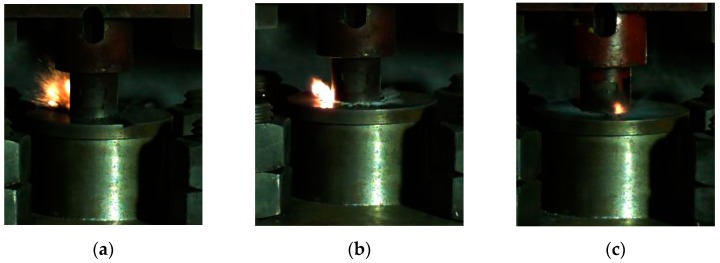
The phenomenon of a drop hammer impacting the reactive material at critical height: (**a**) material No. 1; (**b**) material No. 2; (**c**) material No. 3.

Based on this method, we could obtain the lower limit of the reaction of energetic materials, which was useful for guiding the safe operation of energetic materials during storage and handling. When the drop height was much higher than the reaction threshold of the sample, the material reacted violently with intense fire and noise. Since the reaction occurred relatively quickly, it was necessary to observe it by means of high-speed photography. When the height of the drop hammer was near the lower limit, the reaction of the material became looming. Typical high-speed photography results are shown in Figure 6. If there was fire in the reaction, the sample was judged to have reacted; otherwise, the sample was judged not to have reacted.

## 3. Results, Analysis, and Discussion

### 3.1. Quasi-Static Compression Test

**Figure 7:**
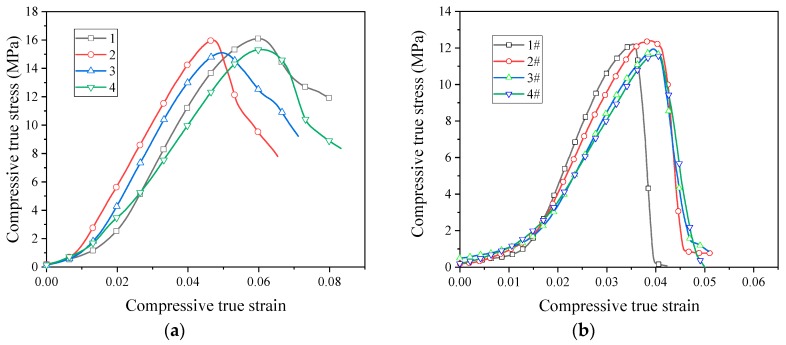
Quasi-static compressive stress–strain curves of unsintered reactive materials: (**a**) micron-sized reactive materials; (**b**) nano-sized reactive materials.

Quasi-static compression curves of unsintered PTFE/Al/CuO reactive materials are shown in Figure 7. It can be seen from the image that the unsintered reactive material was brittle, without yielding and plastic deformation. Moreover, the stress–strain relationship was closely related to the particle size of PTFE. The failure stress of reactive materials prepared using micron-sized PTFE was basically around 15 MPa, and nano-sized PTFE reactive materials were basically stable at around 12 MPa. At this time, the content of the Al/CuO thermite had little effect on the strength of the material.

**Figure 8:**
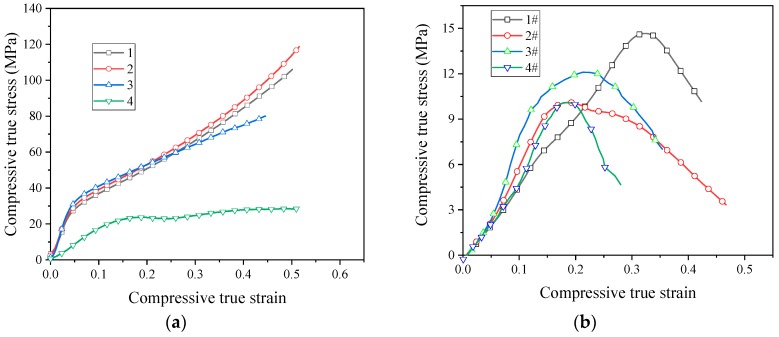
Quasi-static stress–strain curve of sintered reactive materials: (**a**) micron-sized reactive materials; (**b**) nano-sized reactive materials.

The quasi-static stress–strain curve of the sintered PTFE/Al/CuO reactive material is shown in Figure 8. After sintering, the strength of the PTFE/Al/CuO reactive material obviously increased. Figure 8a shows that the sintered micron-sized reactive material exhibited obvious elastic–plastic properties during compression. In particular, materials No. 1 and No. 2 did not fail during quasi-static compression. This indicates that, after sintering, the ductility of micron-sized PTFE reactive materials greatly improved. These materials had no obvious yielding phenomenon, and the stress value when 0.2% plastic strain occurred was taken as yield strength. The yield strength of materials No. 1 to No. 3 increased sequentially, but the strength of No. 4 was significantly lower than the other three materials. This indicates that the addition of Al/CuO thermite at an appropriate amount can increase the strength of the reactive material, while excess Al/CuO thermite reduces the strength of the material. When the content of the Al/CuO thermite reached 25%, the yield strength of the reactive material could be increased to 30.4 MPa. However, the plastic modulus of the reactive material then began decreasing. When the content of the thermite further increased, the elastic modulus of the reactive material also began decreasing. We speculate that this may be because a large amount of Al/CuO thermite promotes the decomposition of PTFE during the sintering process, which causes the material to produce more pores.

The sintered nano-sized reactive materials were still brittle materials, as seen in Figure 8b. After sintering, the strength of the nano-sized PTFE reactive material was significantly lower than that of the micro-sized PTFE reactive material. The strength of the nano-sized reactive material decreased after the addition of the Al/CuO thermite. The use of nano-sized PTFE was not conducive to the strength of the reactive material after sintering.

The stress at the point where the stress began decreasing in the stress–strain curve is defined as the compressive strength. Sintered micron-sized reactive materials did not fail during quasi-static compression, the stress did not decrease. Therefore, we could only obtain their yield strength and modulus. The parameters of the reactive materials obtained from the quasi-static compression test are shown in Table 3.

### 3.2. SHPB Dynamic Compression Test

**Figure 9:**
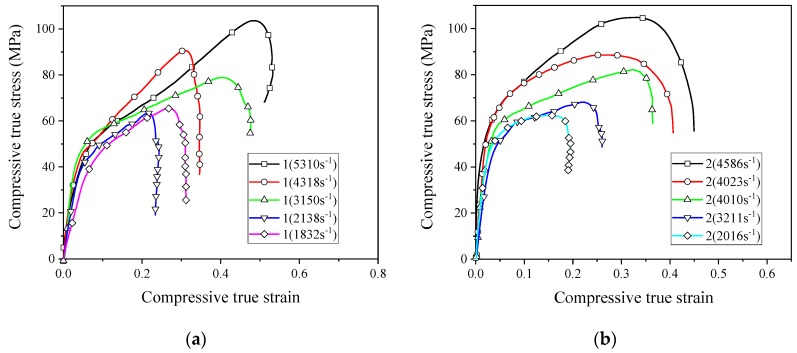

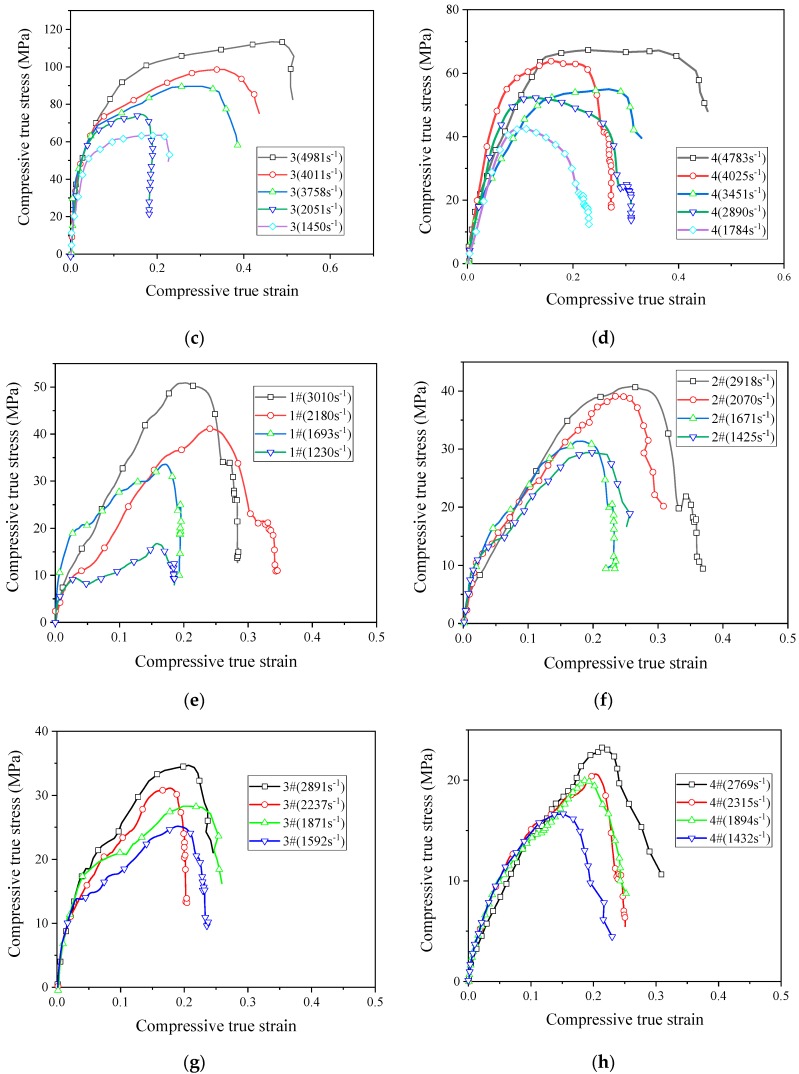
The stress–strain curves of reactive materials at different strain rates; (**a**) Material No. 1; (**b**) material No. 2; (**c**) material No. 3; (**d**) material No. 4; (**e**) material No. 1#; (**f**) material No. 2#; (**g**) material No. 3#; (**h**) material No. 4#.

In order for the reactive material to release energy, performing an impact loading action is required. Therefore, the application of reactive materials is inseparable from the study of its dynamic properties. Based on the SHPB device driven by the high-pressure chamber, the dynamic compressive mechanical properties of reactive materials were studied. Figure 9 shows the stress–strain curves of eight reactive materials during dynamic compression.

The stress–strain curves show that all the eight reactive materials exhibited a positive strain rate effect under dynamic impact. In other words, as the strain rate increased, the failure strength of the reactive material increased. Micron-sized reactive materials also exhibited plastic deformation under dynamic compression, and the nano-sized reactive materials were still brittle. Since many pores were produced in the sample of the nano-reactive material after sintering, the stress–strain curve was not smooth and the stress–strain curve fluctuated greatly, whereas the law was not obvious at the beginning of compression. Among the No. 1 to No. 4 micron-sized reactive materials, the strength of material No. 3 was the highest at similar strain rates. The dynamic compressive strength of the sintered nano-sized reactive material was less than 50% of the micro-reactive material. These laws are consistent with quasi-static compression. In the nano-sized reactive materials, the strength of material 1# was the highest at similar strain rates. Taking the failure strength of reactive materials at a strain rate of about 2900 as an example, the failure strengths of materials 1#, 2#, 3#, and 4# were 50.8 MPa, 40.8 MPa, 34.7 MPa, and 23.2 MPa, respectively. When the strain rate was higher than 1600 s^−1^, a higher content of Al/CuO thermite led to a lower strength of the nano-sized material.

### 3.3. Fitting and Verification of Johnson–Cook Constitutive Model

The Johnson–Cook (JC) constitutive model is an empirical constitutive model which can reflect the strain rate effect and temperature effect of materials. The material parameters measured in the above test could be used to fit the JC (Johnson–Cook) constitutive model [32]. The model is defined as follows:

(8) $$\sigma_{p}=[A+B{\left(\varepsilon_{p}^{}\right)}^{n}][1+C\ln{\dot{\varepsilon }}_{p}^{*}][1-T^{*m}],$$

where $\sigma_{p}$ is the plastic stress, $\varepsilon_{p}^{}$ is the effective plastic strain, ${\dot{\varepsilon }}_{p}^{*}=\dot{\varepsilon }/{\dot{\varepsilon }}_{0}$ is the normalized effective plastic strain rate, ${\dot{\varepsilon }}_{0}=10^{-3}s^{-1}$ is the quasi-static strain rate, and $T^{*}=(T-T_{room})/(T_{melt}-T_{room})$ is the homologous temperature. The five material constants are *A*, *B*, *C*, *n*, and *m*.

**Figure 10:**
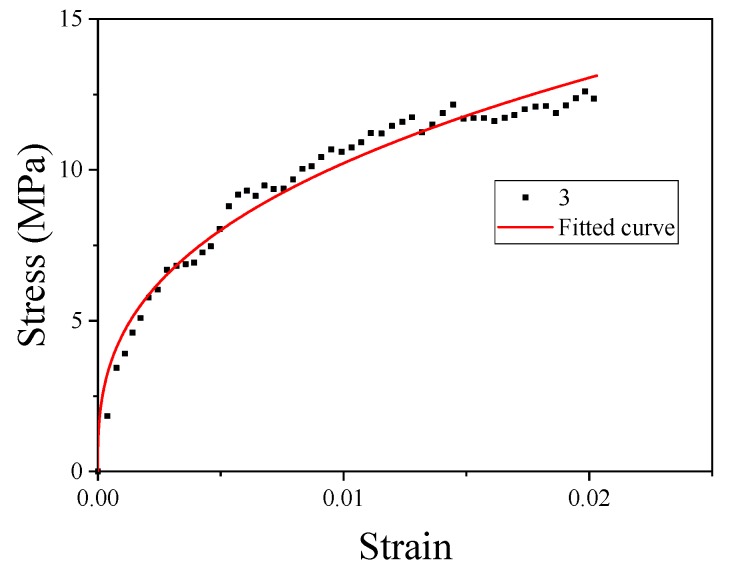
Fitting curves of parameters B and n.

The strength of material No. 3 was relatively high; thus, material No. 3 was selected for JC constitutive fitting. When $T=T_{room}$ and ${\dot{\varepsilon }}_{p}^{*}=1$, the JC model can be written as

(9) $$\sigma_{p}=[A+B{\left(\varepsilon_{p}^{}\right)}^{n}],$$

where the constant *A* is the basic yield stress at low strains. The yield strength of material No. 3 could be obtained from Table 3. The plastic section of the quasi-static curve of material No. 3 was taken to fit *B* and *n*. The fitting curve is shown in Figure 10.

If $T=T_{room}$ and $\varepsilon_{p}=0$, the JC model can be written as

(10) $$\sigma_{p}=A\left(1+C\ln\dot{\varepsilon_{p}^{*}}\right)$$

where $\sigma_{p}$ at this time represents the yield strength of the material measured at different strain rates using the SHPB device. Regardless of the temperature effect, there is only one unknown parameter, *C*, left. Parameter *C* can be obtained by linear fitting. The parameters of reactive material No. 3 are shown in Table 4.

In order to verify the validity of the parameters of the JC model, the SHPB test was simulated by ANSYS/DYNA finite element simulation software. The finite element ANSYS/DYNA software is the world’s leading finite element software for explicit dynamic analysis, which can accurately deal with various highly nonlinear problems.

**Figure 11:**
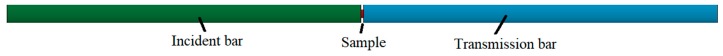
Numerical simulation model.

Firstly, we built a model according to the actual dimensions of the test device and the sample. Both the incident bar and the transmission bar were cylinders with a diameter of 20 mm. The dimensions of the sample were ∅10 mm × 3 mm. In order to avoid interference from the free-end reflected signal, the length of the incident bar was set to 1000 mm, and the length of the transmission bar was set to 400 mm. The final model image is shown in Figure 11.

The software HyperMesh was used to divide the mesh. The mesh elements were all SOLID164. This is an eight-node hexahedron element. The grid size of the cross-section of the bar was 0.4 mm. As the SHPB experiment is a one-dimensional mechanics process in design, the axial mesh could be simplified appropriately. The size of the grid in the axial direction was 1 mm, which reduced the amount of calculation required. The boundary was consistent with reality and was a free boundary. Both the incident bar and the transmission bar were Al bars, which were also defined in the simulation using the JC constitutive model. The model parameters of the bar are shown in Table 5.

**Figure 12:**
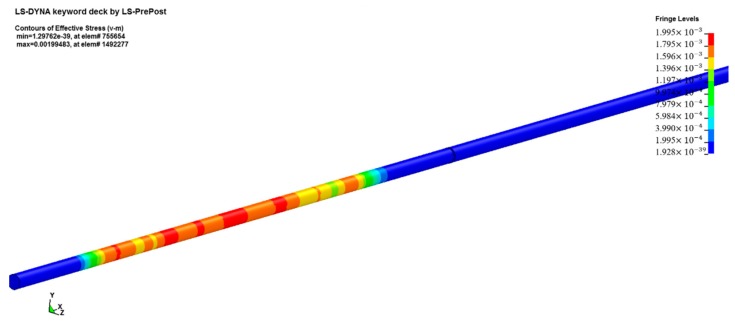
Numerical simulation process.

In the actual SHPB test, the elastic compression pulse wave was generated by the bullet impacting the incident bar. In order to be consistent with the experiment, the compressed wave measured by the experiment was directly loaded into the incident bar in the numerical simulation, as shown in Figure 12.

**Figure 13:**
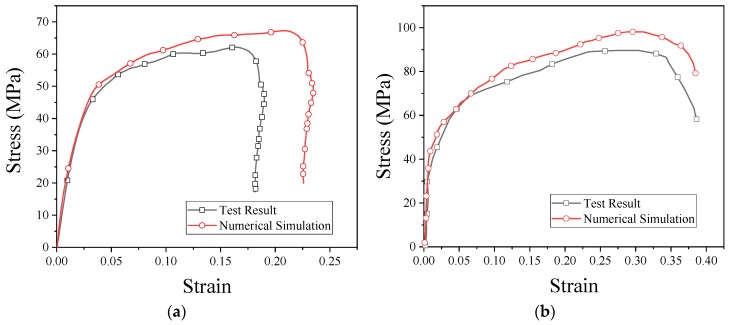
Stress–strain curves obtained from the numerical simulation and SHPB test: (**a**) strain rate of 2051 s^−1^; (**b**) strain rate of 3758 s^−1^.

The stress–strain curves obtained from the numerical simulation and experiment are shown in Figure 13. As can be seen from the figure, the results of the numerical simulation are basically consistent with the experimental results. From these results, it is clear that PTFE/Al/CuO reactive materials can be characterized by the JC model, and the fitted model parameters are reliable. These parameters can be used for other numerical simulation studies of reactive materials.

### 3.4. Drop Hammer Test of PTFE/Al/CuO Reactive Material

**Figure 14:**
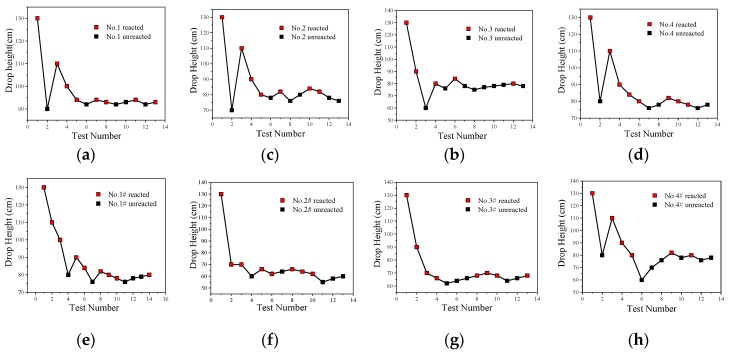
The drop hammer test data points of PTFE/Al/CuO reactive materials; (**a**) Material No. 1; (**b**) material No. 2; (**c**) material No. 3; (**d**) material No. 4; (**e**) material No. 1#; (**f**) material No. 2#; (**g**) material No. 3#; (**h**) material No. 4#.

**Figure 15:**
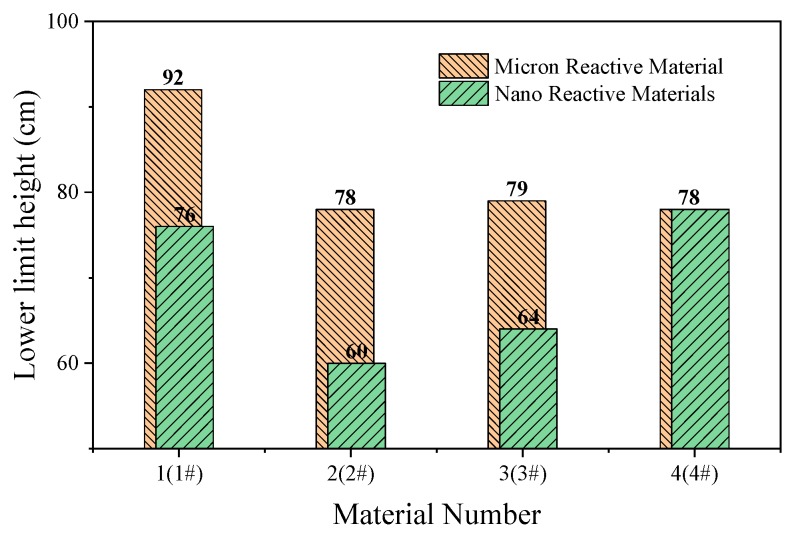
Lower limit height of micron- and nano-sized reactive materials.

The impact-induced reaction characteristics of PTFE/Al/CuO reactive materials were studied using the drop hammer test. The dimensions of the samples were ∅10 ± 0.4 mm × 3 ± 0.2 mm, and the minimum height change interval of the drop hammer was 10 mm. In the experiment, if the reactive material reacted, the height of the drop hammer was lowered, while, if there was no reaction, the height of the drop hammer was increased. Test data and results are shown in Figure 14 and Figure 15, respectively.

The content of Al/CuO thermite in the reactive materials No. 1 to No. 4 was gradually increased from zero. When a small amount of Al/CuO thermite was added to the micro-sized PTFE/Al, the lower limit height of the reactive material was reduced by 25%, which indicates that the sensitivity of the reactive material was increased. However, the lower limit height of reactive materials No. 2 to No. 4 did not change significantly. In general, a larger particle size difference of the materials increases the difficulty of the reaction. In this paper, the particle sizes of micron-sized PTFE/Al and Al/CuO thermite in materials No. 2 to 4 differed by several orders of magnitude. This may explain why the change in the content of Al/CuO thermite did not change the lower limit of the reactive material.

In order to make the Al/CuO thermite play a better role, the micron-sized PTFE/Al was replaced with nanoparticles without changing the proportion of the element of the reactive material. The lower limit height of the nano-sized reactive materials No. 1# to No. 4# changed significantly with the increase in Al/CuO thermite, which was different from the original micron-sized reactive materials. Compared to the micron-sized reactive materials, the lower limit height of the sample was reduced by up to 23% when nano-sized reactive materials were used. With the increase in Al/CuO thermite, the lower limit height of the nano-sized reactive materials firstly decreased and then increased. The test results show that the sensitivity of reactive materials was higher when the content of Al/CuO thermite was between 12.5% and 25%. At this time, the particle size of all the components in the reactive material was similar; thus, the reactive activation conditions of the reaction between CuO and Al in the reactive material were no longer different. The results also indicate that the nano-sized PTFE/Al/CuO reactive materials were easily induced by drop hammer impact when the Al content was high. Therefore, if we want to further reduce the lower limit height of the micro-sized PTFE/Al reactive materials used in the previous section, we should increase the content of nano-sized Al powder in the material.

**Figure 16:**
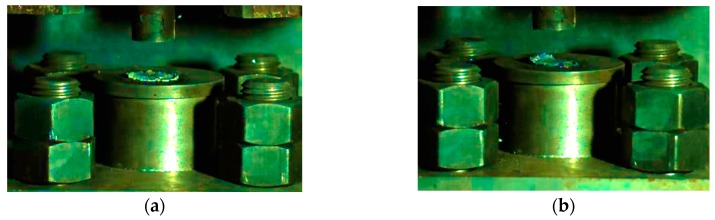

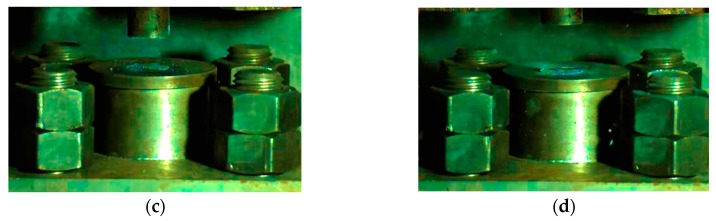
Residue of the reactive material sample: (**a**) reactive material No. 1; (**b**) reactive material No. 3; (**c**) reactive material No. 1#; (**d**) reactive material No. 3#.

The residue of the samples impacted by the drop hammer at the lower limit height is shown in Figure 16.

The residue of sample No. 1 had no Al/CuO thermite and was quite complete and thick, while the residue of sample No. 3 containing Al/CuO thermite was relatively thin and irregular in shape. After adding the Al/CuO thermite, the internal shear friction increases during the impact, which is the main reason for the ignition of the reactive materials [19]. Materials No. 1# and No. 3# were nano-reactive materials. Their residues were significantly thinner and more fragmented than micron-reactive materials, which indicates that the nano-sized material was more susceptible to breakage during impact. As we all know, the nano-sized reactive materials of the same mass have a larger surface area than micron-sized reactive materials. The much larger surface area can significantly alter combustion behavior, as well as ignition behavior, by increasing sensitivity [25]. In addition, the sensitivity of Al/CuO thermite is high and its combustion velocity faster than the PTFE/Al. Therefore, it was easier to initiate a reaction in the new reactive materials compared with the micron-sized PTFE/Al.

**Figure 17:**
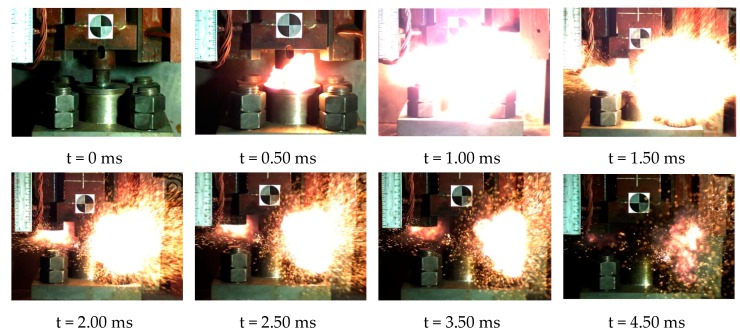
Video frames of reaction process of No. 4 reactive material under drop hammer impact.

When the height of the drop hammer was much higher than the lower limit height of sample, we could observe the violent reaction phenomenon. The reaction process of the reactive material sample could be obtained completely using the high-speed camera (FPS4000). Taking reactive material 4 as an example, the reaction phenomena corresponding to a drop height of 130 cm is shown in Figure 17. At this point, the sample was subjected to a violent impact from the drop hammer, causing a severe chemical reaction. It can be observed that the reactive material was completely crushed before beginning to react, and a large fireball was quickly generated and then scattered. Although the mass of the material was less than 1 g, its energy release was considerable. The reaction duration of reactive material sample No. 4 was about 3.75 ms. Compared with general explosives, this reactive material has a lower reaction rate [33,34,35]. The reaction process of the reactive material was closer to high-speed combustion.

**Figure 18:**
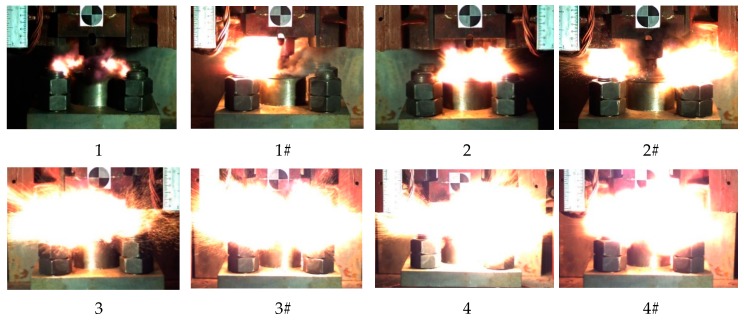
Image of the most violent reaction moment of eight kinds of reactive materials.

The reaction process of eight kinds of reactive materials corresponding to a drop height of 130 cm was recorded and analyzed. The most intense reaction time of all the reactive materials is shown in Figure 18. 

It can be seen from Figure 18 that a higher content of Al/CuO thermite in the reactive materials led to a more intense and larger fire. The flame produced by the sample of the reactive material with 25% and 50% of Al/CuO thermite was very close, and the flame produced by the samples with nano-sized particles was more intense. When the content of Al/CuO thermite was below 25%, the increase in content of Al/CuO thermite could significantly increase the size of the fireball. After the content of Al/CuO thermite exceeded 25%, the change was no longer obvious. Since the energy release efficiency of the reactive material was less than 20% under the impact of drop hammer [27], the size of the fire could reflect the energy release efficiency of reactive material to a certain extent. Therefore, when the content of Al/CuO thermite reached 25%, the energy release efficiency of the reactive material greatly improved. 

In reality, the reactive materials are also affected by various factors, such as different shapes, temperature changes, vibrations, etc. [36,37]. This will be the direction of our future research.

## 4. Conclusions

In this paper, eight kinds of PTFE/Al/CuO reactive material were designed. The mechanical properties, impact sensitivity, and reaction characteristics of the PTFE/Al/CuO reactive materials were studied by quasi-static compression, dynamic compression, numerical simulation, and drop hammer test. By analyzing the test results, the conclusions below can be drawn.

The quasi-static mechanical properties of unsintered PTFE/Al/CuO reactive materials were mainly determined by PTFE. After sintering, with the increase in content of Al/CuO thermite, the strength of the micro-sized PTFE/Al/CuO firstly increased and then decreased. When the content of the Al/CuO thermite was around 25%, the yield strength of the material was maximum. After sintering, the strength of the nano-sized PTFE reactive material was significantly lower than that of the micro-sized PTFE reactive material.

The dynamic compression test showed that the compressive strength of PTFE/Al/CuO reactive materials was strain-rate-sensitive. PTFE/Al/CuO reactive materials could be characterized by the Johnson–Cook constitutive model. The JC model parameters were obtained through experiments, and the PTFE reactive materials could be further studied by numerical simulation.

The nano-sized Al/CuO thermite could effectively improve the sensitivity of PTFE/Al reactive materials. As the Al/CuO thermite was added to the micro-sized PTFE/Al reactive material, the material’s lower limit height was reduced by 25%. Compared with the micro-sized reactive materials, the lower limit height of nano-sized reactive materials was reduced by more than 23%. When the content of Al/CuO thermite was between 12% and 25%, the lower limit height of the nano-sized PTFE/Al/CuO reactive material had a minimum.

Through high-speed camera analysis, it was found that the reaction rate of the reactive material was slower than that of an explosion and was closer to a combustion process. When the content of Al/CuO thermite was below 25%, the increase in Al/CuO thermite content could significantly increase the fire of the material in the reaction. The internal shear friction of the reactive material may be one of the main reasons for initiation.

## Figures and Tables

**Table 1:** Composition of reactive materials.

Number	(73.5%) PTFE + (26.5%) Al (μm) (wt.%)	(73.5%) PTFE + (26.5%) Al (nm) (wt.%)	(81.5%) CuO + (18.5%) Al (nm) (wt.%)
1	100	-	0
2	87.5	-	12.5
3	75	-	25
4	50	-	50
1#	-	100	0
2#	-	87.5	12.5
3#	-	75	25
4#	-	50	50

**Table 2:** Raw materials used in the test. USA—United States of America.

Materials	Code Name	Particle Size	Manufacturer	Production Area
PTFE	7A X	35 μm	DuPont	USA
	A162	350 nm	Xingwang plastic material Co., Ltd.	China Changsha
Al	No-M-001-4	5 μm	Tianjiu metal materials Co., Ltd.	China Changsha
	NO-M-001-1	50 nm	Naiou Nano Technology Co., Ltd.	China Shanghai
CuO	NO-O-003-1	50 nm	Naiou Nano Technology Co., Ltd.	China Shanghai
Coupling agent	KH550	-	Dow Corning	USA

**Table 3:** PTFE/Al/CuO reactive material parameters obtained from quasi-static compression test.

Materials	Yield Strength (MPa)	Elastic Modulus (MPa)	Materials	Compressive Strength (MPa)	Elastic Modulus (MPa)
1	25.9	660.7	1#	14.3	61.2
2	27.8	751.4	2#	12.1	87.1
3	30.4	887.3	3#	15.0	97.3
4	20.1	192.9	4#	9.6	64.4

**Table 4:** Johnson–Cook (JC) model parameters of reactive material No. 3.

Density (g/cm^3^)	Elastic Modulus (MPa)	*A* (MPa)	*B* (MPa)	*n*	*C*
2.79	887.30	30.40	34.32	0.35	6.29 × 10^−2^

**Table 5:** JC constitutive model parameters of metal rod.

Density (g/cm^3^)	Elastic Modulus (MPa)	*A* (MPa)	*B* (MPa)	*n*	*C*
2.7	7.17 × 10^4^	3.65 × 10^2^	4.26 × 10^2^	0.34	1.5 × 10^−2^
